# A Multimodal Imaging–Based Deep Learning Model for Detecting Treatment-Requiring Retinal Vascular Diseases: Model Development and Validation Study

**DOI:** 10.2196/28868

**Published:** 2021-05-31

**Authors:** Eugene Yu-Chuan Kang, Ling Yeung, Yi-Lun Lee, Cheng-Hsiu Wu, Shu-Yen Peng, Yueh-Peng Chen, Quan-Ze Gao, Chihung Lin, Chang-Fu Kuo, Chi-Chun Lai

**Affiliations:** 1 Department of Ophthalmology, Chang Gung Memorial Hospital, Linkou Medical Center Taoyuan Taiwan; 2 College of Medicine Chang Gung University Taoyuan Taiwan; 3 Department of Ophthalmology Keelung Chang Gung Memorial Hospital Keelung Taiwan; 4 Center for Artificial Intelligence in Medicine Chang Gung Memorial Hospital, Linkou Medical Center Taoyuan Taiwan

**Keywords:** deep learning, retinal vascular diseases, multimodal imaging, treatment requirement, machine learning, eye, retinal, imaging, treatment, model, detection, vascular

## Abstract

**Background:**

Retinal vascular diseases, including diabetic macular edema (DME), neovascular age-related macular degeneration (nAMD), myopic choroidal neovascularization (mCNV), and branch and central retinal vein occlusion (BRVO/CRVO), are considered vision-threatening eye diseases. However, accurate diagnosis depends on multimodal imaging and the expertise of retinal ophthalmologists.

**Objective:**

The aim of this study was to develop a deep learning model to detect treatment-requiring retinal vascular diseases using multimodal imaging.

**Methods:**

This retrospective study enrolled participants with multimodal ophthalmic imaging data from 3 hospitals in Taiwan from 2013 to 2019. Eye-related images were used, including those obtained through retinal fundus photography, optical coherence tomography (OCT), and fluorescein angiography with or without indocyanine green angiography (FA/ICGA). A deep learning model was constructed for detecting DME, nAMD, mCNV, BRVO, and CRVO and identifying treatment-requiring diseases. Model performance was evaluated and is presented as the area under the curve (AUC) for each receiver operating characteristic curve.

**Results:**

A total of 2992 eyes of 2185 patients were studied, with 239, 1209, 1008, 211, 189, and 136 eyes in the control, DME, nAMD, mCNV, BRVO, and CRVO groups, respectively. Among them, 1898 eyes required treatment. The eyes were divided into training, validation, and testing groups in a 5:1:1 ratio. In total, 5117 retinal fundus photos, 9316 OCT images, and 20,922 FA/ICGA images were used. The AUCs for detecting mCNV, DME, nAMD, BRVO, and CRVO were 0.996, 0.995, 0.990, 0.959, and 0.988, respectively. The AUC for detecting treatment-requiring diseases was 0.969. From the heat maps, we observed that the model could identify retinal vascular diseases.

**Conclusions:**

Our study developed a deep learning model to detect retinal diseases using multimodal ophthalmic imaging. Furthermore, the model demonstrated good performance in detecting treatment-requiring retinal diseases.

## Introduction

### Background

Retinal vascular diseases, including diabetic macular edema (DME), neovascular age-related macular degeneration (nAMD), myopic choroidal neovascularization (mCNV), and retinal vein occlusion (RVO), highly affect visual function and lead to loss of working ability and impaired life quality [1-4]. Anti–vascular endothelial growth factor (VEGF) can improve visual outcomes for patients with retinal diseases [5]. Early disease detection and timely management can prevent disease progression and advanced visual impairment.

With its advancement in recent years, artificial intelligence has recently been used for several applications in the medical field, including for disease monitoring, diagnosis, and treatment [6]. In ophthalmology, deep learning—an artificial intelligence technique—can potentially detect eye diseases, such as diabetic retinopathy, glaucoma, nAMD, and retinopathy of prematurity, as well as refractive errors [7]. Different ocular pathologies can be identified using different imaging modalities. Multiple imaging modalities are available for retinal vascular disease diagnosis. Although the use of retinal fundus photography for diagnosis is feasible, robust diagnosis may require further imaging, such as through the use of optical coherence tomography (OCT), chorioretinal angiography (ie, fluorescein angiography [FA] and indocyanine green angiography [ICGA]), and optical coherence tomography angiography (OCTA). Deep learning has been applied for various imaging techniques. In addition to color fundus images, which are commonly used for detecting eye diseases [7], other imaging modalities are useful in deep learning–based applications. For example, OCT has been used for diagnosis and referral in patients with retinal diseases [8,9], and OCTA has been used for identifying nonperfusion areas in the retina [10].

### Objective

Multimodal imaging in ophthalmology could improve the accuracy of disease diagnosis. The increased application of multiple imaging modalities for disease detection has led to advancements in deep learning–assisted disease diagnosis. An et al [11] used OCT combined with retinal fundus photography for glaucoma diagnosis. Meanwhile, Vaghefi et al [12] demonstrated an increased accuracy when using multimodal imaging to train an algorithm for OCT, OCTA, and retinal fundus photography for detecting dry AMD. However, little research has investigated the use of deep learning techniques in multimodal imaging for determining retinal vascular diseases. In our study, we developed a deep learning–based model for detecting retinal vascular diseases and diseases requiring anti-VEGF treatment through the use of multimodal retinal imaging, including color fundus photography, OCT, and FA with or without ICGA (FA/ICGA).

## Methods

### Study Participants

In this retrospective study, we included patients who underwent clinical examinations involving retinal fundus photography, OCT, and FA/ICGA from 2013 to 2019 at Chang Gung Memorial Hospital, Linkou Medical Center, Taipei and Keelung branches. The retinal fundus photos were obtained using 1 of the 2 color fundus cameras (Topcon Medical Systems; digital non-mydriatic retinal camera: Canon). OCT was performed using OCT machines (Heidelberg Engineering Inc; Avanti, Optovue Inc), and FA/ICGA images were obtained using fundus angiography machines (Heidelberg Engineering, Inc). The study protocol was approved by the Institutional Review Board of Chang Gung Memorial Hospital (no. 201900477B0), and the study adhered to the tenets of the Declaration of Helsinki.

### Data Classification

In our study, we identified retinal vascular diseases, including DME, nAMD, mCNV, branch retinal vein occlusion (BRVO), and central retinal vein occlusion (CRVO). Patients without a history of anti-VEGF treatment were included. After review of the multimodal images of each eye, disease diagnoses and need for anti-VEGF treatment were determined by 3 trained retinal ophthalmologists (LY, CHW, and SYP, who had 20, 10, and 6 years of clinical experience, respectively). Eye images were first reviewed by 2 of the retinal ophthalmologists (CHW and SYP). The ophthalmologists (CHW and SYP) excluded images with poor quality or nondifferentiable diagnosis. When the disease labels assigned by the ophthalmologists differed, a consensus was reached through discussion among all 3 retinal ophthalmologists. The senior retinal ophthalmologist (LY) again confirmed the image labels that were consistent in the first labeling. The patients were classified into DME, nAMD, mCNV, BRVO, and CRVO groups according to their disease diagnosis. The retinal ophthalmologists further defined diseases as anti-VEGF treatment requiring or non–treatment requiring. Based on the published literature, the treatment requirement was defined separately in each retinal vascular disease according to the features in different images [1,2,13-15]. Moreover, in the control group, we included patients who had undergone retinal fundus photography, OCT, and FA/ICGA examination for clinical purposes, but the examinations revealed no remarkable lesions or only lesions not related to retinal vascular diseases. For multimodal imaging, retinal fundus photos were macular centered; OCT images were fovea centered; and FA/ICGA images, which were randomly selected from different phases, were macular centered.

### Data Management

**Figure 1:**
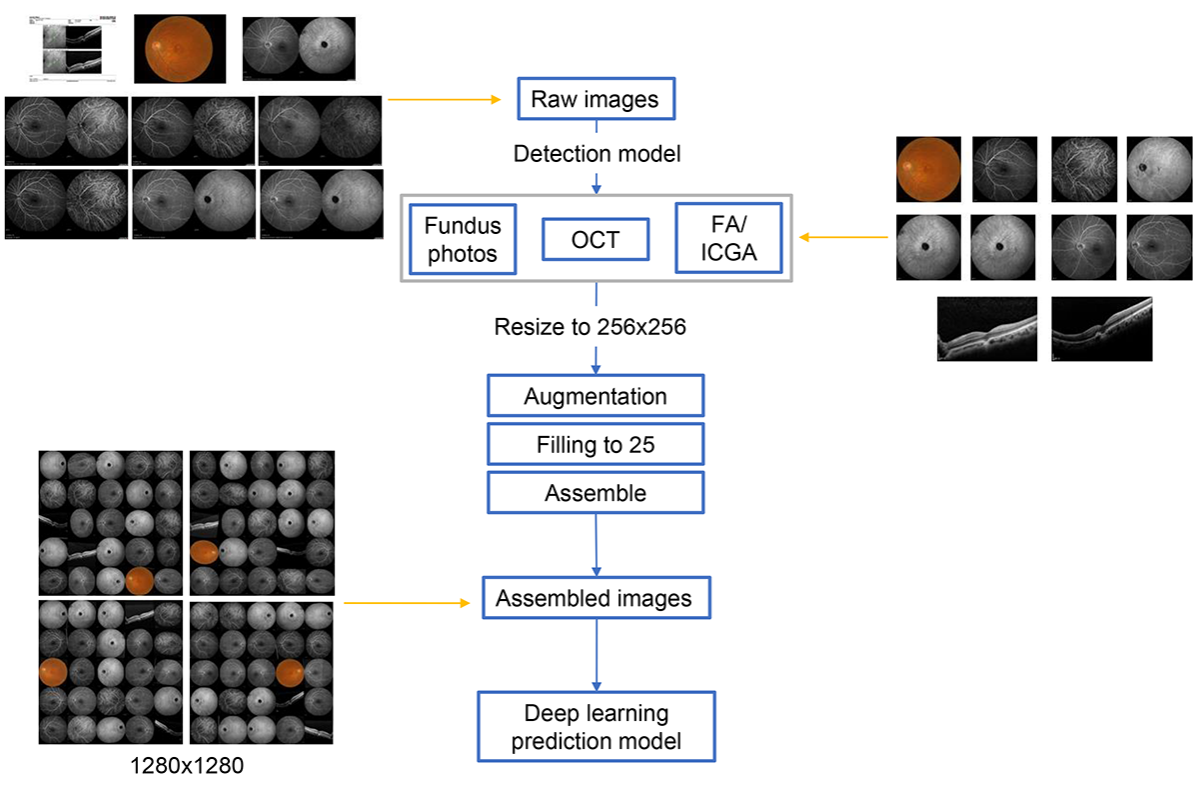
Flowchart of multimodal image management and processing. OCT: optical coherence tomography; FA/ICGA: fluorescein angiography with or without indocyanine green angiography.

The data management and image processing were performed on the same eye. We collected images of retinal fundus photography, OCT, and FA/ICGA from each eye. The flowchart of the image collection process is displayed in Figure 1. First, images were evaluated by the detection model to select and crop for different image types. The detection model, Cascade R-CNN [16], was trained with 599 images in different imaging modalities. The isolated images were first resized to 256 × 256 pixels. Subsequently, isolated images were augmented by slight adjustment of the brightness and contrast level, foggy masking, compression, rotation, horizontal flipping, and the addition of side lines. Then, 25 images were randomly selected from different imaging modalities and assembled. At least one image was required from each imaging modality. The assembled image package consisted of 25 segmented images from the same eye based on a combination of images with various augmentations and components of fundus retinal photography, OCT, and FA/ICGA. The size of the assembled images was 1280 × 1280 pixels, consisting of 25 images with a size of 256 × 256. Then, the image package was sent to the model for prediction.

### Model Architecture

**Figure 2:**
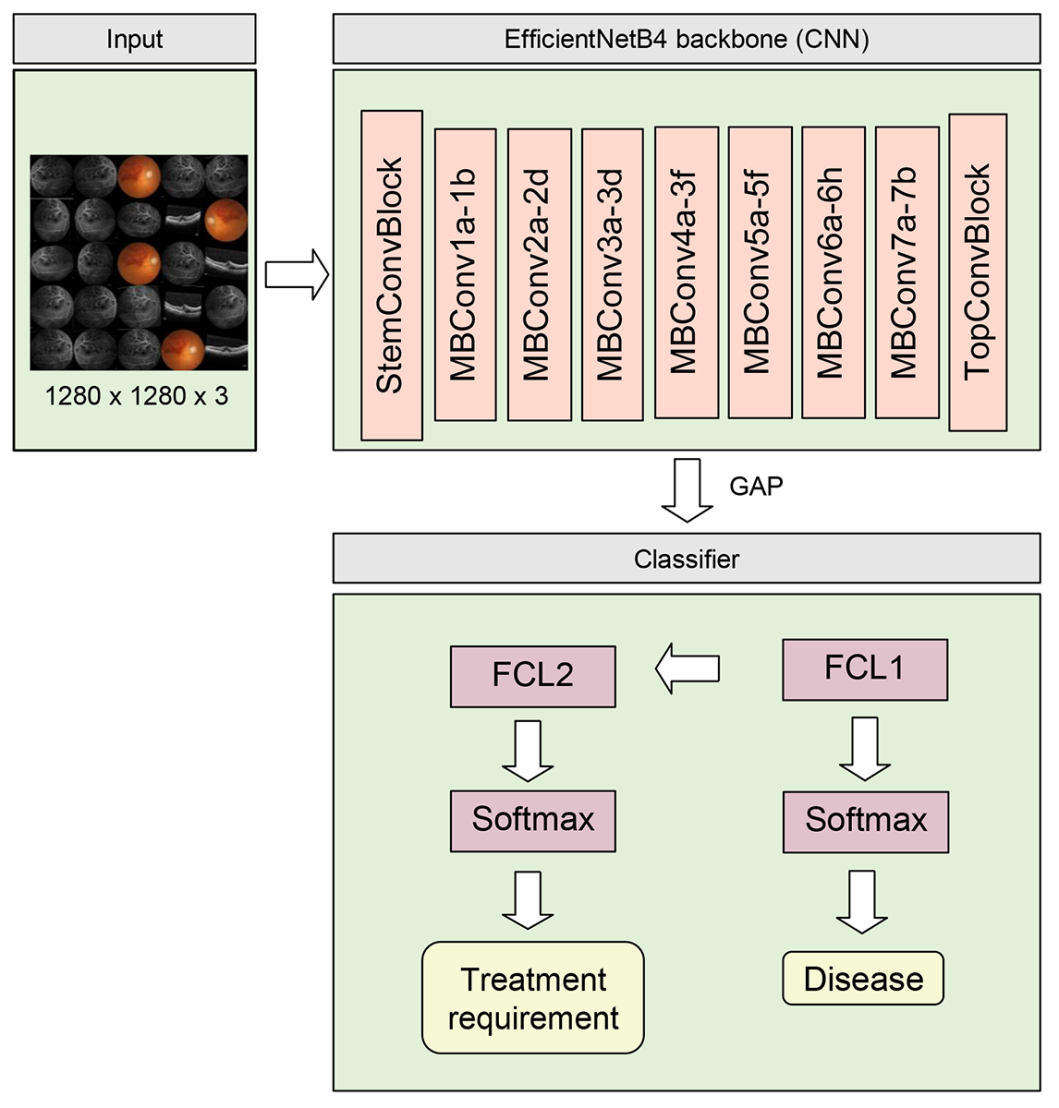
Architecture of the deep learning prediction model. CNN: convolutional neural network; FCL: fully connected layer; GAP: global average pooling.

In our study, EfficientNetB4 was used as the convolutional neural network (CNN) for the classification model (Figure 2). Because our goal was to aid disease diagnosis and the detection of disease severity, the models had 2 outputs: (1) disease classification and (2) treatment requirement determination. However, features indicating severity may differ based on the disease. Our model first delivered disease prediction for differentiating different retinal vascular diseases. We then designed a layer consisting of a fully connected, reshaped, and weighted sum to facilitate the model classification of treatment requirement partially according to the results from the disease prediction part. In addition, to visualize the features for model prediction, heat maps were generated using gradient-weighted class activation mapping [17], which used the gradient based on the output scores to show the activation map for the specific image. The features of the heat maps were highlighted in a lighter color.

### Model Training

Image packages were split into training, validation, and testing data sets in a 5:1:1 ratio, respectively. The model was trained based on noisy student [18] pretrained weight and optimized using an AdamW optimizer [19]. The model was trained 3 times with different combinations of training and validation data sets. We also tested different parameters including learning rates of 1e-4, 1e-5, and 5e-5, and batch sizes of 8, 12, and 16. Subsequently, the model with the best performance in the training and validation data sets was selected and evaluated in the testing data set (Multimedia Appendices 1 and 2). The learning rate and batch size were set as 5e-5 and 16, respectively. Data preprocessing and the training and evaluation of the model were completed on a NVIDIA DGX-1 server with the Ubuntu 18.04 operating system. Image preprocessing, including conversion, augmentation, and assembly, was conducted using ImageMagick 7.0.10 [20]. Images were evaluated and cropped using Mmdetection 1.0.0 [21] and Pytorch 1.4.0 [22], and the bounding box was labeled using CocoAnnotator [23]. Tensorflow 2.2 [24] was used as the framework to train and evaluate the deep learning model.

### Statistical Analysis

Receiver operating characteristic (ROC) curves were used for differentiating different retinal vascular diseases and treatment-requiring diseases, and the area under the curve (AUC) was measured for each ROC curve. Moreover, the sensitivity, specificity, and accuracy of the model were calculated. Regarding model performance in predicting different retinal diseases, the AUC, sensitivity, specificity, and accuracy were based on a one-versus-rest comparison. Additionally, a confusion matrix was created and demonstrated sensitivity in disease prediction. Statistical analysis was performed using the Sklearn 0.23.2 package in Python (Python Software Foundation).

## Results

### Study Participants and Data Distribution

In total, 2992 eyes of 2185 patients were included in our study. In the first labeling of 2992 eyes, 212 (7.08%) were differently labeled by CHW and SYP, and a consensus was reached after discussion among all 3 retinal ophthalmologists. Among the 2780 eyes with consistent labels in the first step, 144 (5.18%) eyes had different labels after review by LY, and a consensus was reached after discussion among all 3 retinal ophthalmologists. The distribution of the included eyes is shown in Table 1.

**Table 1:** Number of eyes included in the control and disease groups.

Groups	Total	Treatment-requiring	Non–treatment-requiring
Control	239	N/A^a^	N/A^a^
DME^b^	1209	788	421
nAMD^c^	1008	809	199
mCNV^d^	211	56	155
BRVO^e^	189	144	45
CRVO^f^	136	101	35
Total	2992	1898	855

^a^N/A: not applicable.

^b^DME: diabetic macular edema.

^c^nAMD: neovascular age-related macular degeneration.

^d^mCNV: myopic choroidal neovascularization.

^e^BRVO: branch retinal vein occlusion.

^f^CRVO: central retinal vein occlusion.

The control, DME, nAMD, mCNV, BRVO, and CRVO groups consisted of 239, 1209, 1008, 211, 189, and 136 eyes, respectively. Among all the disease groups, 788, 809, 56, 144, and 101 eyes required treatment in the DME, nAMD, mCNV, BRVO, and CRVO groups, respectively. Subsequently, 2138, 427, and 427 eyes were assigned to the training, validation, and testing data sets, respectively. We used 5117 retinal fundus photos, 9316 OCT images, and 20 922 FA/ICGA images, and the distribution of the images in different data sets is shown in Table 2.

**Table 2:** Distribution of image number used in different modalities for different data sets.

Modality	Total (n=2992)	Training (n=2138)	Validation (n=427)	Testing (n=427)
Retinal fundus photos	5117	3662	709	746
OCT^a^	9316	6704	1272	1340
FA/ICGA^b^	20922	14932	2959	3031

^a^OCT: optical coherence tomography.

^b^FA/ICGA: fluorescein angiography with or without indocyanine green angiography.

### Model Performance

**Figure 3:**
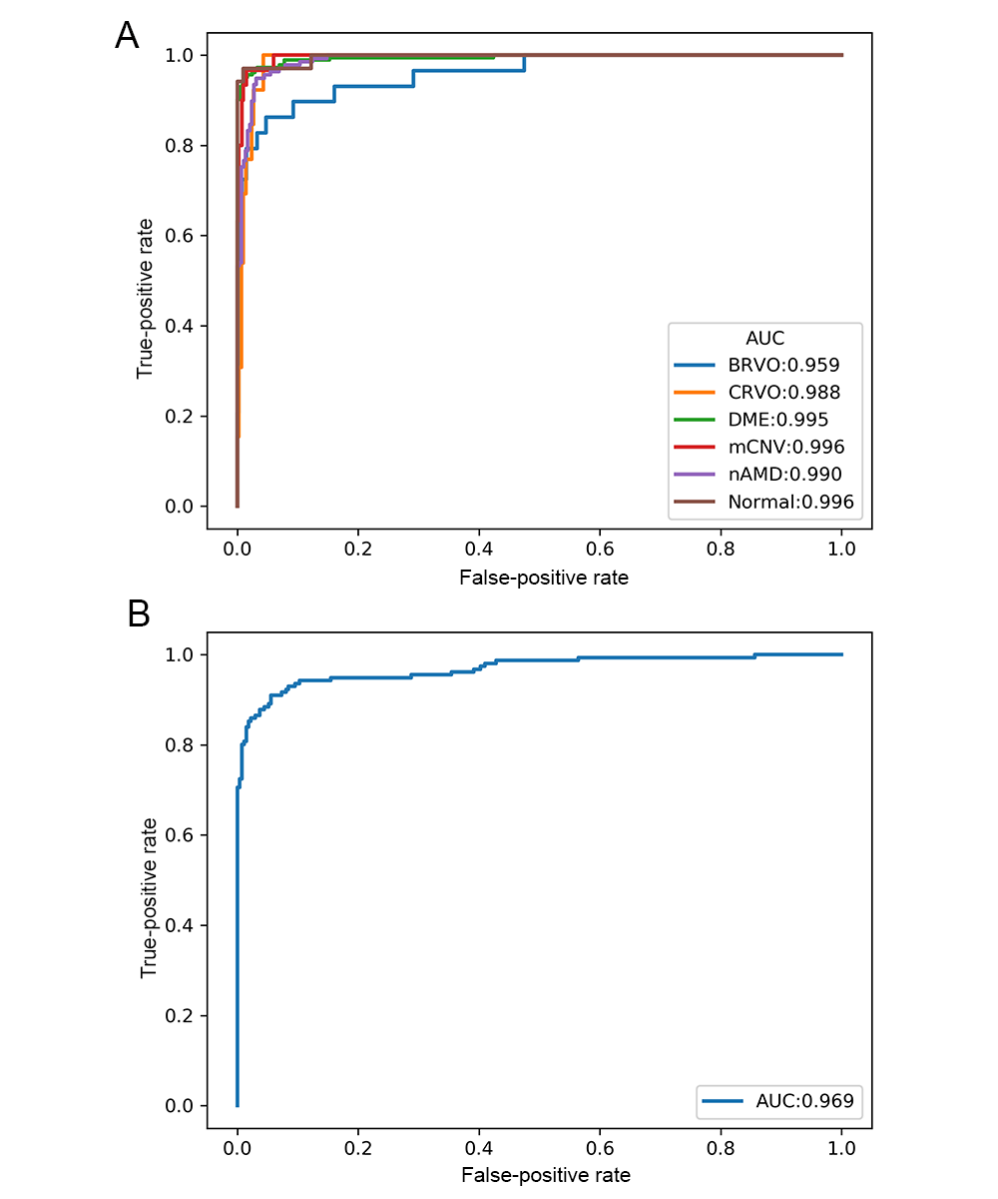
Receiver operating characteristic curves of the model performance for (A) predicting different retinal vascular diseases and (B) identifying treatment-requiring diseases. AUC: area under the curve; BRVO: branch retinal vein occlusion; CRVO: central retinal vein occlusion; DME: diabetic macular edema; mCNV: myopic choroidal neovascularization; nAMD: neovascular age-related macular degeneration.

**Figure 4:**
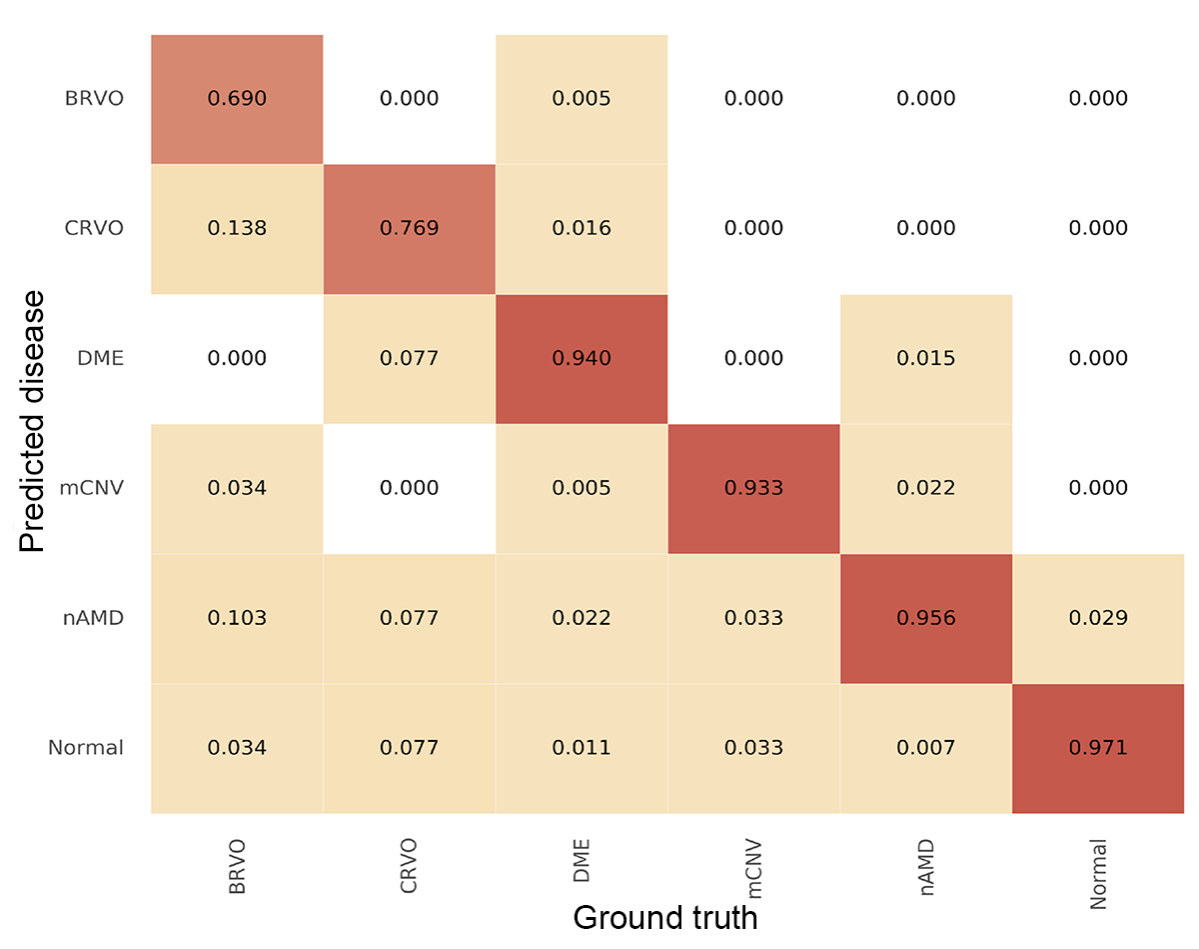
Confusion matrix demonstrating the performance of the prediction model in different retinal vascular diseases. BRVO: branch retinal vein occlusion; CRVO: central retinal vein occlusion; DME: diabetic macular edema; mCNV: myopic choroidal neovascularization; nAMD: neovascular age-related macular degeneration.

Model performance was evaluated using the testing data set. ROC curves are illustrated in Figure 3, and the AUC for each curve was determined. For disease identification, the overall AUC was 0.987, and the AUC was the highest in the mCNV (0.996) and control (0.996) groups, followed by the DME (0.995), nAMD (0.990), CRVO (0.988), and BRVO (0.959) groups. For predicting diseases requiring anti-VEGF treatment, the AUC was 0.969. Details regarding the model sensitivity and specificity are provided in Table 3. For retinal vascular disease prediction, the sensitivity was the highest for the control (0.971) group, followed by the nAMD (0.956), DME (0.940), and mCNV (0.933) groups, whereas the sensitivity of RVO identification was the lowest (0.690 for BRVO and 0.769 for CRVO). Regarding the prediction of diseases requiring anti-VEGF treatment, the sensitivity was 0.904 and specificity was 0.945. The accuracy for disease prediction was the highest in the control and mCNV (0.984) groups, followed by the BRVO and CRVO (0.977), DME (0.967), and nAMD (0.963) groups. The accuracy for the detection of treatment-requiring diseases was 0.930. The confusion matrix is shown in Figure 4.

**Table 3:** Sensitivity, specificity, and accuracy of the model in the prediction of retinal vascular diseases and treatment-requiring diseases.

Value	Sensitivity	Specificity	Accuracy
Control	0.971	0.985	0.984
DME^a^	0.940	0.988	0.967
nAMD^b^	0.956	0.966	0.963
mCNV^c^	0.933	0.987	0.984
BRVO^d^	0.690	0.997	0.977
CRVO^e^	0.769	0.983	0.977
Treatment requirement	0.904	0.945	0.930

^a^DME: diabetic macular edema.

^b^nAMD: neovascular age-related macular degeneration.

^c^mCNV: myopic choroidal neovascularization.

^d^BRVO: branch retinal vein occlusion.

^e^CRVO: central retinal vein occlusion.

### Heat Maps for Model Prediction

**Figure 5:**
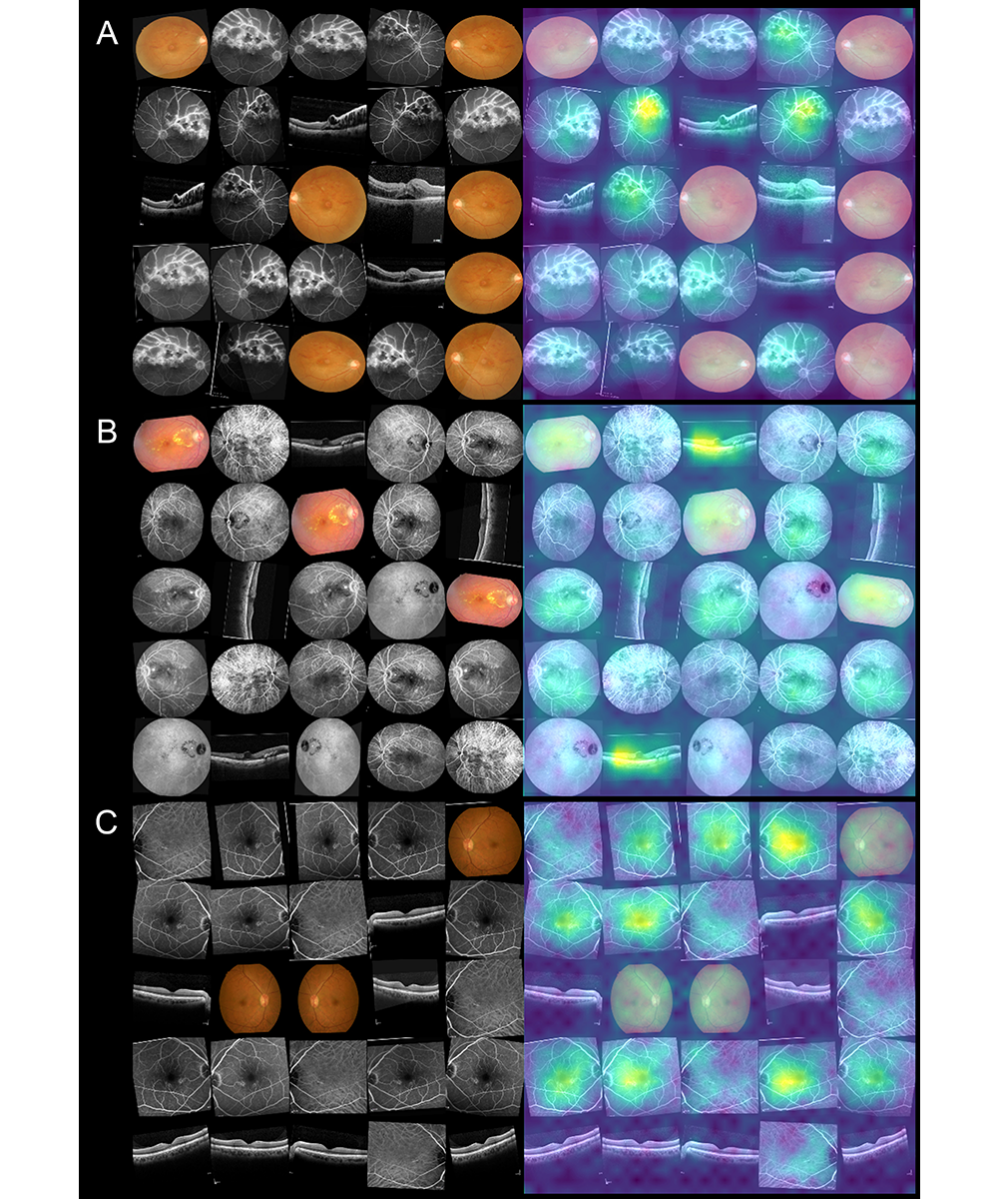
Sample heat maps generated by the prediction model in a true-positive patient with (A) treatment-requiring branch retinal vein occlusion, (B) treatment-requiring diabetic macular edema, and (C) non–treatment-requiring age-related macular degeneration.

Heat maps for visual explanations of our model predictions were generated using gradient-weighted class activation mapping, and the samples are shown in Figure 5. In the heat maps, the model could simultaneously identify the lesion in different imaging modalities. Regarding different retinal vascular diseases, the model had different weights in different image modalities. For example, in eyes with RVO, the model highlighted the exudates and hemorrhage dot in retinal fundus photos, ischemic area, and leaking point in FA/ICGA. In patients requiring treatment for DME, the model highlighted retinal vessels within the macula in retinal images, the central swelling area in OCT images, and the leaking or staining lesions in FA/ICGA images.

## Discussion

### Main Findings

In this study, we used multimodal imaging to develop a deep learning–based model for the prediction of retinal vascular diseases, including DME, nAMD, mCNV, BRVO, and CRVO, and to determine whether anti-VEGF treatment was required. This model had average AUCs of 0.987 and 0.969 for predicting retinal vascular diseases and for predicting treatment-requiring diseases, respectively. The heat map shows that the model can identify disease features through multimodal retinal imaging.

### Ophthalmology Imaging in Deep Learning

Previous studies have proven the efficacy of using different image modalities in deep learning–based models for predicting retinal diseases. In addition to retinal fundus images for identifying diabetic retinopathy, AMD, and glaucoma [7], a deep learning model using OCT for retinal layer segmentation and retinal disease identification was developed by the DeepMind group [8]. Moreover, deep learning could help to detect ischemic zones in retinal vascular diseases through the use of ultra-wide-field FA [25]. The aforementioned studies demonstrated that deep learning can be effectively applied for a single retinal imaging modality. However, few investigations have been conducted to study the application of deep learning models for predicting diseases using more than one retinal imaging modality. OCT and retinal fundus images have been used concomitantly for dry AMD [26] and glaucoma [11] diagnosis. However, previous studies have either used a single imaging modality or focused on predicting a single retinal disease. To date, few studies have evaluated the performance of deep learning models with multimodal retinal imaging for predicting multiple retinal vascular diseases.

### Multimodal Imaging–Based Deep Learning Model for Retinal Vascular Diseases

To our knowledge, this is the first study to use multimodal deep learning–based architecture for detecting multiple retinal vascular diseases. In our study, we used multiple image modalities, including retinal fundus photography, OCT, and FA/ICGA, for predicting neovascular retinal diseases, including DME, nAMD, mCNV, and RVO [27]. Furthermore, this model can identify diseases requiring anti-VEGF treatment. In clinical settings, multimodal retinal images are crucial for ophthalmologists to treat retinal diseases. Occasionally, a feature in a retinal image modality may be shared by many retinal diseases. For example, increased central retinal thickness in OCT can be present in DME, nAMD, mCNV, and RVO, but retinal fundus images may vary among these diseases. The features of nAMD and mCNV may appear similar in retinal fundus images, and an ICGA is needed for differentiating them [2]. Therefore, multimodal imaging is required for the diagnosis and treatment determination of different retinal diseases [28]. Our model with multimodal imaging was similar to real-world ophthalmology practice with regard to the diagnosis for multiple retinal diseases and determination of disease severity. In real-world practice, the model may help with the screening of the diseases and treatment-requiring status, saving ophthalmologist’s time and effort on reviewing the images. Although the AUC of different retinal vascular diseases demonstrated excellent differentiation, defined as AUC > 0.8 [29], the RVO groups showed relatively low sensitivity. This might be related to the low number of eyes used for model training. In the future investigation, the generative adversarial network may be implemented to synthesize ophthalmic images and solve the problem of an inadequate number of images [30].

### Detection of Treatment-Requiring Retinal Vascular Diseases

Because expenses involved in using anti-VEGF drugs in the treatment of retinal vascular diseases are high, patients being administered these drugs may need to meet strict criteria to claim reimbursement from insurance companies in many regions [31]. In Taiwan, the use of intravitreal anti-VEGF treatment for DME, nAMD, mCNV, and RVO requires prereview by members of the Taiwan National Health Insurance program for reimbursement [32,33]. An efficient and accurate method for evaluating a patient’s retinal vascular disease status and disease severity may be essential. Our model could not only aid ophthalmologists in disease diagnosis and in determining the need for anti-VEGF treatment for retinal vascular diseases but also help with the prereview of anti-VEGF treatment.

### Image Variability for the Model

The model developed in the present study is highly flexible in terms of image input. It does not depend on a fixed image distribution for different modalities. The only requirement is at least one image for each imaging modality. We investigated the model accuracy for packages with different numbers of images, and 25 images in a 5 × 5 matrix had the highest performance. Moreover, we tested different CNN models and different compositions of imaging modalities to determine which could achieve the highest accuracy (Multimedia Appendix 3). Using the CNN of EfficientNetB4 with images of retinal fundus photography, OCT, and FA/ICGA had the best performance. The images from the same eye can be randomly arranged or augmented during the preprocessing stage before being used in the prediction model. The visualized heat maps show that the model has the ability of simultaneous differentiation of retinal diseases with the use of different imaging modalities. With DME, for example, both the central retina in OCT and the leaking points in FA had high weightage. For BRVO, the model highlights areas with hemorrhage in retinal fundus images, increased retinal thickness in OCT images, and nonperfusion in FA images. These findings are compatible with the clinical features of retinal diseases [34,35] and indicate that our model produces reasonable and reliable predictions of retinal vascular diseases.

### False Prediction of the Model

**Figure 6:**
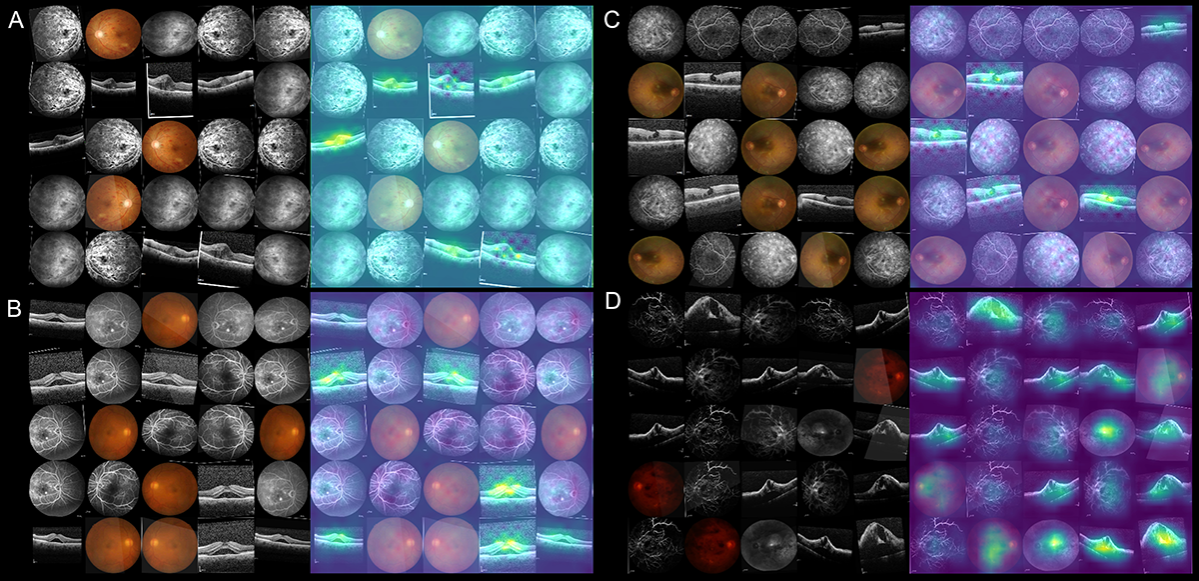
Sample heat maps for false prediction of the model: (A) false prediction of treatment-requiring diabetic macular edema (DME) in a patient with coexisting DME and central retinal vein occlusion (CRVO); (B) false prediction of treatment-requiring age-related macular degeneration (AMD) in a patient with central serous chorioretinopathy; (C) false prediction of treatment-requiring DME in a patient with epiretinal membrane, lamellar macular hole, and diabetic retinopathy; (D) false prediction of treatment-requiring DME in a patient with advanced CRVO.

Regarding false predictions of retinal diseases, sample heat maps are presented in Figure 6. We observed that the model provided wrong predictions mostly for eyes with advanced-stage diseases or coexisting retinal diseases. Retinal vascular diseases may share undistinguishable features in advanced stages. For example, in an advanced stage of a disease, retinal hemorrhage, retinal nonperfusion, and macular edema could appear to have the same prominence in CRVO as in DME and advanced diabetic retinopathy [36,37]. The coexistence of diabetic retinopathy with DME may produce clinical features similar to those of RVO with macular edema [38]. Additionally, other retinal disorders, such as central serous chorioretinopathy, may display features similar to those of retinal vascular diseases and lead to misdiagnosis by the model. As for diseases requiring anti-VEGF treatment, false prediction was noted in cases with borderline disease activity or other retinal disorders, such as central serous chorioretinopathy and epiretinal membrane, for which anti-VEGF treatment is not indicated.

### Study Limitations

This study had some limitations. First, the model requires the use of multiple image modalities, including OCT and FA/ICGA, which some eye-care facilities may not be equipped with. Although the study focused on deep learning–based prediction with multimodal imaging, clinical application may require more investigation. Second, images used in the study underwent quality checks. The efficacy during application to a real-world clinical setting may be affected by the patient’s condition and the image quality [39]. Additionally, some ocular diseases affecting image signal transmission could affect image quality and retinal disease diagnosis [40,41]. Third, images from different machine manufacturers not included in our study might have affected the model accuracy. A transfer learning approach could be adopted in cases where images are obtained from different machine manufacturers. Fourth, we did not consider other retinal vascular diseases, such as retinal neovascularization caused by uveitis or infection. The model is inapplicable to diseases not included in our study. Fifth, we only identified disease statuses that may require anti-VEGF treatment. Disease statuses requiring other treatments, such as laser therapy, were not analyzed in the current study. Furthermore, images of the most advanced disease stages with features such as severe vitreous hemorrhage or diffused chorioretinal atrophy would have been excluded due to nondifferentiable diagnosis. Sixth, a relatively small number of eyes in the RVO groups led to decreased accuracy in disease prediction and more data may be needed for better model performance. Last, the study group only included patients without previous anti-VEGF treatment. The accuracy in patients with a history of anti-VEGF treatment needs further investigation.

### Conclusions

We developed a deep learning–based model using multimodal imaging for predicting retinal vascular diseases and determining whether anti-VEGF treatment is required. This model can facilitate the differentiation of DME, nAMD, mCNV, BRVO, and CRVO and help in determining the indication for anti-VEGF treatment.

## Appendix

Multimedia Appendix 1 Splitting of the data sets and training process of the model.

Multimedia Appendix 2 Performance of the model trained with different (A) learning rates and (B) batch sizes. AUC: area under the curve.

Multimedia Appendix 3 Performance of the model with different convolutional neural networks and composition of imaging modality. AUC: area under the curve; CF: color fundus photography; CNN: convolutional neural network; FA/ICGA: fluorescein angiography with or without indocyanine green angiography; OCT: optical coherence tomography.

## Abbreviations

AUC: area under the curve
BRVO: branch retinal vein occlusion
CNN: convolutional neural network
CRVO: central retinal vein occlusion
DME: diabetic macular edema
FA: fluorescein angiography
mCNV: myopic choroidal neovascularization
nAMD: neovascular age-related macular degeneration
OCTA: optical coherence tomography angiography
ROC: receiver operating characteristic
VEGF: vascular endothelial growth factor
