# Supplementary figures and images for: A Multimodal Imaging–Based Deep Learning Model for Detecting Treatment-Requiring Retinal Vascular Diseases: Model Development and Validation Study
Source: JMIR Med Inform. 2021 May 31;9(5):e28868. doi: 10.2196/28868 (PMC8204240; doi:10.2196/28868)

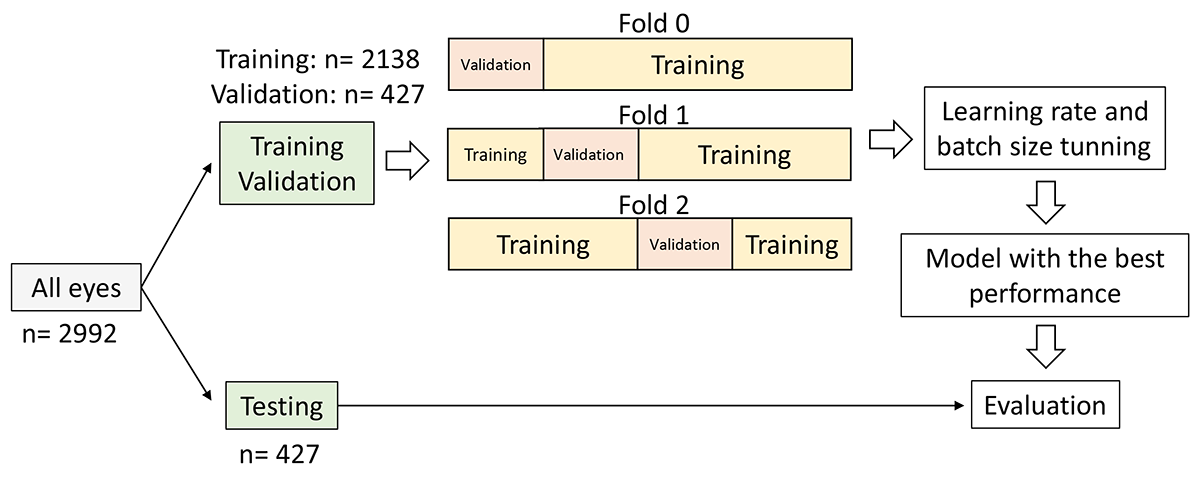

Supplement: Multimedia Appendix 1 [file medinform_v9i5e28868_app1.png]

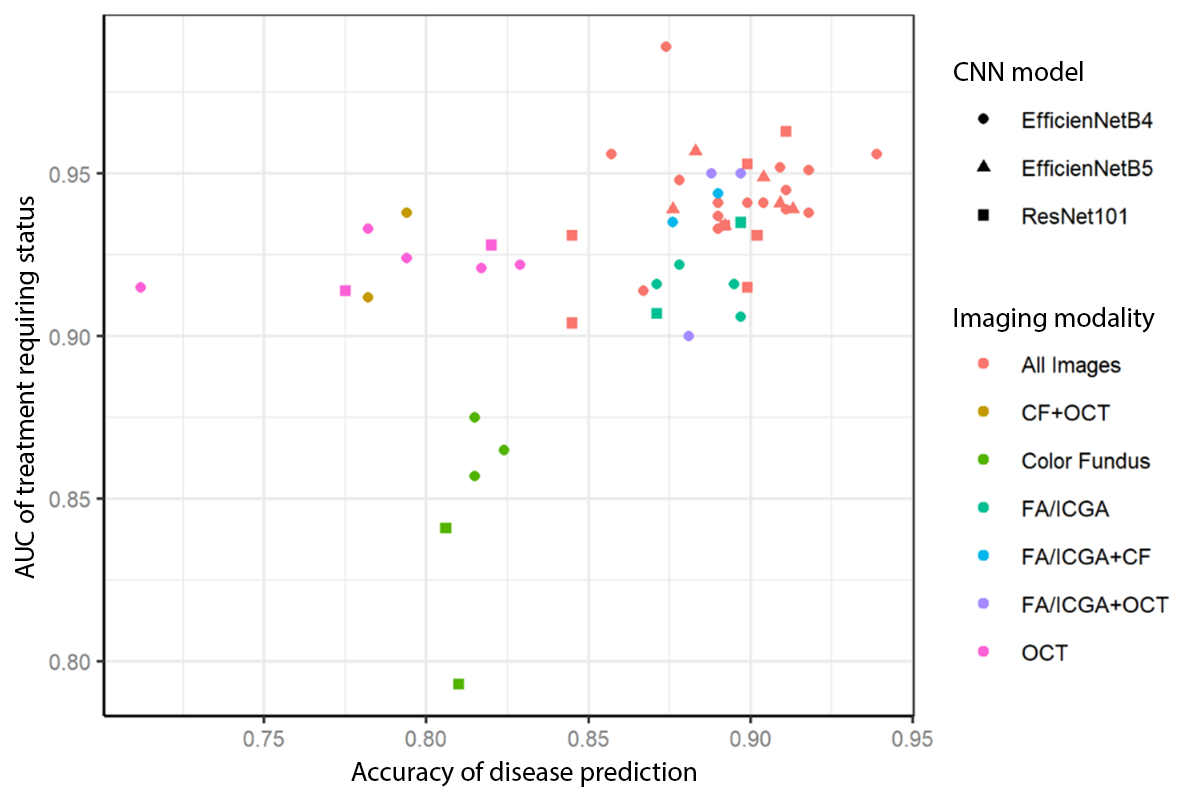

Supplement: Multimedia Appendix 3 [file medinform_v9i5e28868_app3.png]
