# Supplementary material for: A Multimodal Imaging–Based Deep Learning Model for Detecting Treatment-Requiring Retinal Vascular Diseases: Model Development and Validation Study
Source: JMIR Med Inform. 2021 May 31;9(5):e28868. doi: 10.2196/28868 (PMC8204240; doi:10.2196/28868)
Supplement: Multimedia Appendix 2 [file medinform_v9i5e28868_app2.pdf]

(A)

Fold	Learning Rate	Batch Size	Disease Accuracy	Severity AUC
0	5.e-5	16	0.902	0.955
**1**	**5e-5**	**16**	**0.927**	**0.967**
2	5e-5	16	0.902	0.966
0	1e-4	16	0.906	0.965
1	1e-4	16	0.909	0.957
2	1e-4	16	0.89	0.951
0	1e-5	16	0.869	0.954
1	1e-5	16	0.895	0.96
2	1e-5	16	0.864	0.942

(B)

Fold	Learning Rate	Batch Size	Disease Accuracy	Severity AUC
0	5e-5	8	0.759	0.907
1	5e-5	8	0.892	0.959
2	5e-5	8	0.834	0.891
0	5e-5	12	0.838	0.948
1	5e-5	12	0.892	0.959
2	5e-5	12	0.899	0.955
0	5e-5	16	0.902	0.955
**1**	**5e-5**	**16**	**0.927**	**0.967**
2	5e-5	16	0.902	0.966
